# Correlation between Coronary Artery Disease with Other Arterial Systems: Similar, Albeit Separate, Underlying Pathophysiologic Mechanisms

**DOI:** 10.3390/jcdd10050210

**Published:** 2023-05-11

**Authors:** Alexandru Achim, Orsolya Ágnes Péter, Mihai Cocoi, Adela Serban, Stefan Mot, Alexandra Dadarlat-Pop, Attila Nemes, Zoltan Ruzsa

**Affiliations:** 1 Department of Cardiology, “Niculae Stancioiu” Heart Institute, University of Medicine and Pharmacy “Iuliu Hatieganu”, Motilor 19-21, 400001 Cluj-Napoca, Romania; 2 Department of Cardiology, Medizinische Universitätsklinik, Kantonsspital Baselland, Rheinstrasse 26, 4410 Liestal, Switzerland; 3 Department of Internal Medicine, Invasive Cardiology Division, University of Szeged, Semmelweis u. 8, 6725 Szeged, Hungary; 4 Vascular Surgery Department, University of Szeged, 6720 Szeged, Hungary

**Keywords:** coronary artery disease, atherosclerosis, carotid artery disease, peripheral artery disease, arterial tree, vulnerable plaque

## Abstract

Atherosclerosis is a multifactorial systemic disease that affects the entire arterial tree, although some areas are more prone to lipid deposits than others. Moreover, the histopathological composition of the plaques differs, and the clinical manifestations are also different, depending on the location and structure of the atherosclerotic plaque. Some arterial systems are correlated with each other more than in that they simply share a common atherosclerotic risk. The aim of this perspective review is to discuss this heterogeneity of atherosclerotic impairment in different arterial districts and to investigate the current evidence that resulted from studies of the topographical interrelations of atherosclerosis.

## 1. Introduction

Coronary artery disease (CAD) is essentially connected with the development of atherosclerotic plaques along the course of coronary arteries, a phenomenon that is still poorly understood despite multiple pathophysiologic contributors. The widely acceptable mechanism for coronary atherosclerosis entails the following stages: endothelial dysfunction and the subendothelial accumulation of low-density lipoprotein (LDL); the oxidization of LDL; the migration of monocytes to the subendothelial level and conversion to macrophages; foam-cell formation; smooth muscle cell multiplication; and finally the apoptosis of foam cells, leading to the formation of necrotic cores. This represents a complex and heterogeneous process requiring decades to show macroscopic evidence in the form of plaque. Several systemic risk factors, such as hyperlipidemia, hypertension, hereditary factors, and vascular features defining wall shear stress and other blood flow hemodynamic factors all contribute to the multifaceted process of plaque production [1].

These factors are expected to have a constant influence on the vascular wall across the vascular tree. However, the fact that atherosclerotic plaques tend to impact specific locations of the arterial tree heightened the significance of local features in the onset and progression of atherosclerosis. The association between coronary artery tree topology and atherosclerotic disease has generated interest in recent years, with several studies looking into various aspects of this interaction [2]. Patients with multiple vascular diseases such as myocardial infarction, stroke, or established peripheral artery disease (PAD) have double the risk of further cardiovascular death, myocardial infarction, or stroke versus patients with either prior myocardial infarction, prior stroke, or established PAD alone [3]. Moreover, the widespread activation of inflammatory cells across the one vascular bed, regardless of the location of the culprit stenosis during an acute event, challenges the concept of a single vulnerable plaque by a rather general inflammatory vascular state [4]. Atherosclerosis is no longer regarded as a simple lipid storage condition, but rather a systemic inflammatory disease, with an identifiable correlation across different arterial locations, since the shared disease is likely to be impacted by similar systemic factors. However, the level of interaction and concordance between the various vascular systems remains unknown because a homogenous “vulnerable patient” phenotype does not exist. In the coronary circulation, for example, some patients present with single vessel disease, whereas others present with multivessel disease, with varied distribution and plaque burden severity. Similar findings have been described in the carotid circulation, with the majority of patients presenting with unilateral disease versus the minority presenting with bilateral disease [5]. Acute coronary syndrome is induced by local thrombosis from a ruptured or eroded plaque, while severe carotid stenosis causing hypoperfusion is significantly predictive of stroke, although this effect may be time-limited [6]. Moreover, carotid atherosclerosis develops later in life compared to coronary disease [7]. Therefore, the heterogeneous atherosclerotic effect on the arterial vascular system could be more than just “two sides of the same coin”.

## 2. Coronary and Carotid Artery Disease

The coronary arterial system and extracranial carotid arteries are the two vascular regions that are implicated in the majority of cardiovascular events. The rupture or erosion of the plaque is thought to be the starting point of the thrombotic (in coronary arteries) or embolic (in carotid arteries) cascades that lead to myocardial infarction or ischemic stroke in both carotid and coronary arteries. The prevalence of coexisting coronary and carotid artery disease ranges from 2 to 14%, with 8% of patients with a history of coronary artery bypass grafting having a severe stenosis in an extracranial carotid artery [8]. Conversely, significant coronary artery stenoses occur in nearly one third of patients with high-grade carotid stenosis who are being considered for carotid surgery [9]. In general, the artery wall thickens during the first radiologically observable phases as a result of an infiltration of foam cell histological alterations in the intima layer. Even though the biology of atherosclerotic process is similar, there are differences in plaque morphology and characteristics. Indeed, plaque erosion, calcified nodules, fibrous cap thickness, and macrophage accumulation may be different in the setting of coronary and carotid artery disease. A large meta-analysis of over 20,000 patients looking at the relationship between coronary and carotid atherosclerosis found that (1) carotid intima-media thickness was increased in a linear manner proportional to the severity of CAD; (2) the carotid plaque presence and calcification were less prevalent, the lipid rich necrotic core was higher, and the intraplaque hemorrhage did not differ in nonsignificant compared with significant CAD; (3) carotid intima-media thickness correlated with the number of diseased coronary vessels; (4) carotid and coronary stenosis and calcification of the 2 systems moderately correlated together; and (5) carotid intima-media thickness ≥ 1.0 mm rather than plaque presence were the best predictors of CAD [10]. These findings highlight the fact that although the pathological elements of atherosclerosis are the same in all arterial beds, the phenotypic picture is not identical between the carotid and coronary systems.

**Figure 1:**
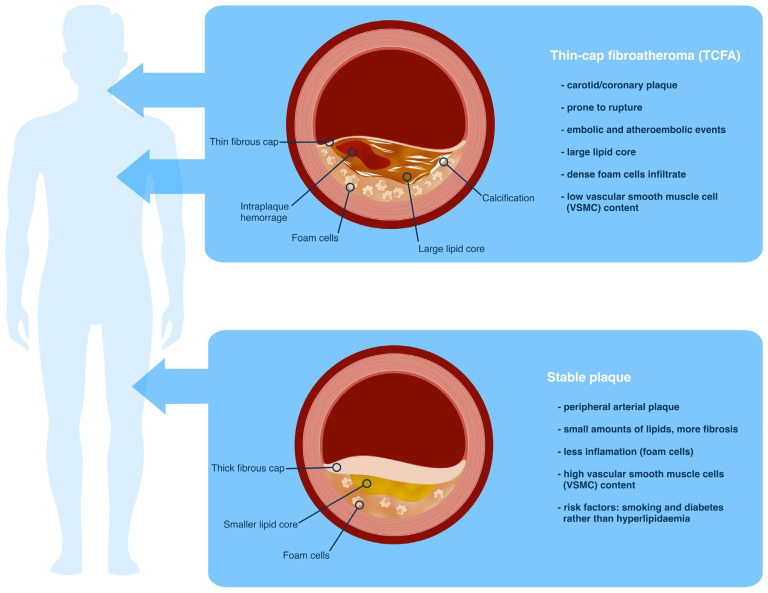
The morphological differences between clinically manifest atherosclerotic plaques located in different areas of the arterial system.

From a pathological perspective, the morphological differences between carotid and coronary plaques follow the same principles towards vulnerability, but with nuances. The term “thin-cap fibroatheroma” (TCFA) was coined from studies of ruptured coronary lesions in which the only distinguishing morphological characteristic was the absence of a luminal thrombus as well as a thin intact nondisrupted fibrous cap. The term is now used in both arterial districts (Figure 1). A difference between coronary TCFAs and carotid TCFAs would be that the same prone-to-rupture plaque composition determines different clinical manifestations: embolic events for carotid TCFAs and rupture and thrombotic occlusion for coronary TCFAs. A histological evaluation of the plaque burden in coronary arteries demonstrated that 70% of plaque ruptures had more than 75% cross-sectional luminal area stenosis, a quarter had narrowing of 50% to 75%, and only 5% were stenosed less than 50% [11]. In contrast, only 40% of TCFAs presented more than 75% luminal stenosis, approximately 50% of lesions were associated with 50% to 75% stenosis, and 10% had less than 50% stenosis. Moreover, the overall plaque burden was greater in plaque rupture populations, and the necrotic core area was larger in ruptured than in TCFAs. Nevertheless, fibrous cap thickness was the best morphological parameter for distinguishing TCFAs from ruptures. Although not as effective as fibrous cap thickness, the degree of macrophage infiltration was useful in distinguishing TCFAs from ruptured plaques [11]. Vulnerable carotid plaques as compared by coronary plaques are characterized by a thicker fibrous cap as compared to those in coronary vessels; a higher incidence of intraplaque hemorrhage; a lower incidence of plaque erosion; and a higher incidence of calcified nodules [12]. However, the largest-ever study of symptomatic carotid plaque histology found that the mechanisms of plaque instability in the carotid circulation remain similar to those in the coronary circulation: a high prevalence of cap rupture, a large lipid core, and dense macrophage infiltrate [13]. Diabetes mellitus is a major risk for calcified and lipid-rich necrotic core carotid plaques as well [14,15], and the use of glucose lowering medication is associated with more fibrose-stable plaque phenotypes [16].

While stable angina and symptoms of CAD are associated with the significance of coronary stenosis [17], patient mortality is mainly affected by acute coronary events for which the incidence is not associated with the degree of stenosis. Indeed, a relevant proportion of unstable plaques have less than 50% stenosis [18]. As a response, one essential concern would be why, after a lengthy time of dormancy, plaques gain vulnerable characteristics such as thrombus development, abrupt lumen obstruction, and ischemia symptoms. TCFAs typically have large ‘late’ necrotic cores with an overlaying thin intact fibrous cap formed primarily of collagen type I and varied degrees of macrophages and lymphocytes, as well as a scarcity or absence of smooth muscle cells. In the coronary artery, fibrous cap thickness <65 µm is considered as a pathological indicator of lesion vulnerability [19], while carotid vulnerable plaques are defined as having a thin fibrous cap thickness of <165 µm with associated macrophage infiltration (CD68 positive macrophages, greater than 25 per high power field) in the absence of plaque rupture [20]. For carotid plaques, the histological characteristic that was most commonly correlated with both cap rupture and duration since stroke was macrophage infiltration, confirming the importance of inflammatory cellular infiltration as a marker of plaque instability and highlighting the potential of targeted imaging for the identification of vulnerable plaques [13]. Thus, the risk stratification of patients with carotid disease, as well as the possibility that anti-inflammatory therapeutic approaches, could perhaps stabilize plaques [13]. Currently, antithrombotic and lipid lowering therapies are the mainstay of medical treatment. Table 1 shows the ubiquitous indication of the guidelines for different arterial locations. Such guidelines also recommend active training and lifestyle modification as being at least as important (Class Ia recommendation) [21].

Besides these morphological differences, there are also rheological differences between the carotid arteries and the other peripheral arteries. Wall shear stress, a local risk factor of atherosclerosis, is decreased in common carotid arteries with evidence of stenosis than in plaque-free carotid arteries [24]. A study by Spring et al. showed that wall shear stress of the common carotid artery is decreased in patients with symptomatic PAD [25]. Other factors such as central pressures, arterial stiffness (pulse wave velocity), and wave reflections can promote atherosclerosis in different arterial beds. Dammers et al. illustrated that mean shear stress differs between the brachial artery and the common carotid artery [26]. In coronary arteries, the atherosclerotic plaques are mainly located along the inner side of the curved coronary arteries due to its particular flow pattern and hydraulic factors originating from inherent epicardial curvatures (coronary blood flow is intermittent and shows wide phasic variations as a result of the systolic contraction of the heart) [27]. Thus, the value of wall shear stress is subject-specific and vessel-specific. Since information on arterial pulsatile hemodynamics can be obtained through non-invasive methods, it may be useful in predicting the occurrence of future cardiovascular events in both primary and secondary settings.

## 3. Coronary and Peripheral Artery Disease

Patients suffering with PAD have a higher risk of subclinical CAD and are at a higher risk of cardiovascular events when compared with healthy individuals [28,29]. The diagnosis of symptomatic PAD has been associated with a 70 percent increased risk of cardiovascular incidents and an 80 percent increased risk of death when compared with patients without PAD [30]. PAD also predicts more extensive coronary disease, such as left main CAD or complicated CAD, as measured by a high SYNTAX score [31]. Unfortunately, many patients with PAD and an ankle-brachial index <0.9 are asymptomatic (more than half in a recent study reported by Lisowska et al.) [32]. Interestingly, the same study showed a much higher prevalence of carotid artery disease in the same population of randomly selected Polish subjects [32].

Age affects the anatomic distribution of atherosclerotic obstructive disease in the distal aorta and iliofemoral vessels. Aortoiliac disease is the most prevalent location of atherosclerosis in patients under the age of 40. In contrast, femoropopliteal pathology accounts for more than 65% of anatomic locations that cause claudication symptoms in adults over the age of 40 [33]. About two-thirds of iliac disease patients will have stenoses, whereas two-thirds of femoral disease patients will have occlusions, the majority of which will be lengthy segmental occlusions [33].

The pathological features of the plaques in the peripheral vascular beds may be heterogeneous even if atherosclerosis and its risk factors are shared by all districts. An in vivo investigation comparing plaque morphology and vascular remodeling in coronary and peripheral arteries, including the carotid, renal, and iliac arteries, was conducted using gray-scale and radiofrequency intravascular ultrasonography [34]. The study found that fibroatheromatous plaques were more common in the coronary arteries and were associated with positive remodeling regardless of the arterial location [34]. Another study found the differential expression of genes in atherosclerotic plaques in different arterial systems [29]. This may contribute to the variations in plaque morphology [35]. Histologically, common findings in PAD include fibroproliferative plaques with low lipid content and a high vascular smooth cell (VSMC) content, as well as a low density of vasa vasorum, fewer inflammatory cells, and more durable plaques [36]. This specific plaque structure increases restenosis following revascularization interventions [36]. Ultimately, in contrast to acute coronary events wherein the underlying pathology is atherothrombosis, the cause of acute limb ischemia in patients with PAD includes in situ thrombosis, emboli from heart and proximal vessels, and graft occlusion.

From a pathological perspective, a study on 239 arteries from amputated limbs found significant atherosclerosis to be present in approximately two-thirds of femoral and popliteal arteries, and in only 40% of infrapopliteal arteries [37]. In other words, in 30% of femoro-popliteal arteries and 60% of infrapopliteal arteries, atherosclerosis was minimal [37]. One-fourth of the arteries with luminal stenosis of less than 70% were stenosed as a result of severe atherosclerosis without thrombi. Just one-third of the remaining thrombi had considerable atherosclerosis associated with them, and the remaining three-fourths had nonsignificant atherosclerosis. These thrombi caused luminal compromise in the remaining cases [37]. Over 70% of all arteries showed medial calcification, with increased extent in the infrapopliteal segment (OR 3.35, *p* = 0.0006); moreover, 60% of patients had calcification in the small arteries of the subcutaneous tissue with varying degrees of luminal fibrosis and occlusion [37]. These results are consistent with other studies, emphasizing the concept of high prevalence of nonatheromatous lesions in PAD and the relative low percentage of lipids in the atheromatous lesions [38,39]. Clinically, this leads to the slow progression of stenosis, the gradual worsening of clinical manifestations, and the simultaneous creation of new collaterals, which are different features compared to coronary or carotid plaques.

As a matter of fact, plaques with high collagen and low muscle cell composition were related with the development of restenosis [40]. As a result, the superficial femoral artery has considerably greater rates of restenosis after endarterectomy than the common femoral artery [41]. Unstable and inflammatory plaques with a high macrophage density and a substantial lipid core were linked to decreased restenosis. Vascular inflammation following balloon angioplasty or stent placement has been recognized as an essential aspect of the restenotic process, and numerous markers of inflammation have been discovered as possible outcome predictors. Inflammation in the artery wall caused by balloon trauma or stent implantation promotes hypertrophic neointima development via vascular smooth muscle cell (VSMC) proliferation and negative vascular remodeling [42]. The formation of neointima and repeated lumen narrowing has been described as a sign of an inflammatory wound healing response exhibited uniquely in vascular tissue [43]. Furthermore, the fibrotic features of a femoral plaque have been linked to constrictive remodeling (arterial lumen reduction) [44].

The degree of inflammation within atherosclerotic plaques varies depending on the location, and this factor plays a significant role in determining the stability and fragility of the plaque. A study comparing the density of lymphocytes and macrophages in the atherosclerotic plaques of the femoral and carotid arteries revealed that the carotid arteries had substantially more severe inflammation than the femoral arteries did [45]. The inflammation of atherosclerotic plaques was also studied using positron emission tomography (PET-CT), and showed that fluorodeoxy-glucose (FDG) uptake measured by the target to background ratio was significantly higher in carotid plaques than in femoral plaques (2.9 ± 0.4 vs. 1.8 ± 0.3, *p* < 0.05) [46]. In fact, another study looking at limb vascular inflammation by measuring the transfemoral gradients of neutrophil myeloperoxidase (MPOx) content and interleukin-6 (IL-6) found that the coexistence of PAD did not necessarily imply a more severe coronary atherosclerosis in patients with CAD, and only those with an inflammatory status of the affected limb presented a more severe CAD [47]. Although the cross-sectional character of this study precludes drawing firm conclusions about causality, its data raises the plausible hypothesis that peripheral vascular inflammation may play a pathogenetic role in CAD by interfering with the coronary endothelial function—findings confirmed in other studies as well [48,49].

In contrast to coronary arteries, where unstable atherosclerotic lesions frequently cause thrombotic occlusions of the affected artery, atherosclerotic plaques in the limb, and other peripheral arteries due to their fibrotic structure, smaller content of lipids and a lower density of inflammatory cells (macrophages that have phagocytized lipid material or foam cells), are more stable and less vulnerable (Figure 1) [50]. Consequently, peripheral sudden thrombotic occlusions of non-stenotic atherosclerotic lesions and embolic complications are much less frequent than in coronary arteries, and aortic, iliac, and femoral plaques can result in infrapopliteal arterial compromise and limb ischemia due to the more frequent occurrence of the embolic compromise of the distant regions.

As a result, a combination of imbricated direct and indirect causative factors connected to the onset of PAD and its hemodynamic consequences are most likely the causes of the increased unfavorable cardiac events in PAD. It is plausible that PAD is actually a marginal manifestation of significant CAD that has already developed, for which preventive interventions may be less beneficial because medical treatment alone does not reduce the increased morbidity risk of PAD [51]. In addition to risk factor profiles that intersect, PAD may independently drive negative cardiovascular outcomes. Between PAD and CAD, phenotypic variations in risk factor profiles are acknowledged. Patients with PAD, for instance, frequently have decreased HDL cholesterol and greater triglyceride levels [30]. Functional limitations in locomotion brought on by PAD may also prevent individuals from participating in cardioprotective activities such as exercise. Additionally, aberrant peripheral vasodilation and paradoxical vasoconstriction in response to elevated metabolic demands during stress are documented in PAD patients. An increased systemic afterload may result from this failure of arterial vasodilation [52]. The modulation of systemic inflammation will be crucial in improving the therapeutic choices for both disease states as a modern understanding of atherosclerotic disease becomes more sophisticated.

Smoking is a strong independent risk factor for PAD [53]. A systematic review demonstrated that that half of PAD cases were due to smoking [54]. In fact, cigarette smoking impacts all phases of atherosclerosis, from endothelial dysfunction to acute clinical events, and it affects all vascular beds. In the carotid artery, smoking has been associated with a consistent increase in intimal-medial thickness [55]. Current smokers were more likely to have either soft or calcified carotid plaques, and former smokers were at greater risk of only echodense carotid plaques when compared to never smokers [56]. In the coronary bed, an in vivo intravascular ultrasound study comparing coronary plaques in smokers vs. non-smokers, cigarette smoking was associated with a higher burden of the necrotic core in the atherosclerotic plaques [57]. Moreover, in an optical coherence tomography study, persistent smoking was associated with an attenuated effect of statin therapy on plaque stabilization in acute coronary syndrome patients [58].

## 4. Other Arterial Systems

**Figure 2:**
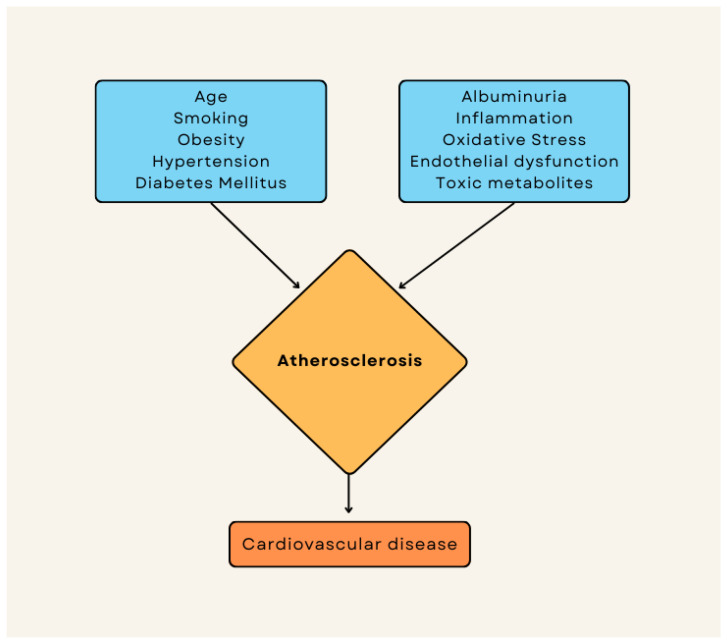
Additional renal risk factors (right panel) contributing to an accelerated atherosclerosis in chronic kidney disease patients.

The progression of chronic kidney disease is associated with the progression of atherosclerosis [59]. The association between renal artery stenosis and CAD is well known, but this harmful interplay is more complex than just a simple causality. For example, renal artery stenosis is the cause of ischemic nephropathy and is an important cause of secondary hypertension. Moreover, patients with end-stage renal disease who are on dialysis due to renal artery stenosis have significantly poorer survival rates than patients dialyzed due to other causes [59]. On the other hand, end-stage renal disease determines diffuse arterial mediocalcinosis, which is associated with significant morbidity and mortality [60]. Additionally, intimal calcification also occurs in patients with renal disease that is associated with atherosclerotic plaques [60]. However, while there is a consistent association between chronic kidney disease and a higher burden of coronary artery calcification [61,62], the associations among phosphorus, calcium, and parathyroid hormone with coronary artery calcification in these patients is inconsistent [63,64]. Nevertheless, there are specific features of atherosclerosis in chronic kidney disease. Other pathogenic pathways are possible, and they may be represented in new risk factors for chronic kidney disease and lead to accelerated atherosclerosis. These factors are illustrated in Figure 2. Chronic kidney disease has been linked to an increase in VLDL (very-low-density lipoprotein) particle accumulation, a decrease in LDL particle size, and alterations in the cholesterol and triglyceride content of LDL and HDL (high-density lipoproteins; which gain triglycerides and lose cholesterol) [65]. Moreover, individuals with chronic renal disease have higher immune-mediated inflammatory activity than equivalent control groups without chronic kidney disease. Increased circulating CRP and cytokine concentrations, an activated phenotype of circulating monocytes (e.g., CD14 + CD16+ monocytes) and resident vascular cells, and the increased synthesis of inflammation-triggered reactive oxygen species are all proinflammatory changes in patients with advanced renal disease [66], and CRP levels were linked with the presence of plaque burden in chronic kidney disease patients [67]. Moreover, it has recently been demonstrated that high urea levels facilitate the posttranslational modification of proteins through a process called protein carbamylation, altering their structure and function, and that chronic inflammation and oxidative stress (which are implicated in the process of atherogenesis) are mechanistically linked to the promotion of protein carbamylation [68].

The NEFRONA study results clinically corroborated these conclusions [67,69]. This study discovered a higher frequency of plaques in advanced chronic kidney disease groups. The presence of plaques in the limb arteries, for example, is strongly linked to chronic kidney disease [70]. These findings imply that the severity of chronic kidney disease is an independent factor in subclinical atheromatosis. The NEFRONA study also demonstrated that the source of chronic kidney disease influences the prevalence of atheromatosis. Diabetic nephropathy, for example, increases the likelihood of developing subclinical atheromatosis [67,69]. With the increased prevalence of PAD in chronic kidney disease and its link to increased mortality in dialysis patients, screening for and the early detection of PAD is critical.

Other arterial systems have been correlated with CAD up to a certain level. Radial artery calcification has been associated both with coronary calcification and significant CAD that required revascularization [71]. This might seem natural in the context of systemic atherosclerosis that diffusely affects the arterial system, but this interesting study raises the issue of screening patients with possible CAD whose radial artery is evaluated for other purposes, either with duplex ultrasound or directly during arterial puncture [72,73]. Another clinical applicability for prior knowledge of the morphological state of the arterial artery is in complex transcatheter interventions that require larger arterial sheaths [74]. It is therefore understood that the correlation of the different arterial systems with each other, in addition to the sophisticated pathophysiological explanations, also have simple and very useful practical considerations, especially in the medical specialties that intersect with cardiovascular pathologies.

In a group of patients undergoing surgery for chronic mesenteric ischemia, 33% had concomitant CAD [75]. Investigators have reported angiographic prevalences of subclavian stenosis of 13% in populations of patients with CAD [76]. Recently, an artificial intelligence (AI) algorithm was successfully applied to validate a deep learning system for coronary CT angiography-derived measures of plaque volume and stenosis severity, demonstrating high performance when compared with expert readers, intracoronary angiography, and intravascular ultrasound [77]. The identification of subclinical atherosclerosis and its predictive impact is a demanding area to investigate and applicate clinically. It is important to remember that while finding subclinical atherosclerosis may be useful in risk stratification, there was no evidence that such identification translated into a better outcome; in fact a carotid study showed a benefit for such early detection recently [78]. Tissue factor, which is an essential predictor of the thrombogenicity of human atherosclerotic lesions, is also abundant in lipid-rich atherosclerotic plaques [79]. These findings suggest that the morphology of atherosclerotic lesions should be considered when selecting preventative and therapeutic approaches in people at risk for atherosclerotic cardiovascular events in the future. Magnetic resonance has the potential to explore the structure of atherosclerotic lesions in vivo. According to one study, magnetic resonance T2 mapping can be used to precisely evaluate plaque lipid concentration noninvasively. Despite a similar degree of luminal stenosis, symptomatic plaques had higher lipid contents than asymptomatic plaques [80]. This innovative approach may be useful in determining the best treatment for atherosclerotic plaques and for monitoring treatment response.

In summary, the physical properties and durability of atherosclerotic plaques vary across the vascular tree. These discrepancies are most likely attributable to haemodynamic forces, vasa vasorum density, and significant diversity in the sensitivity of different regions of the arterial tree to various atherosclerosis risk factors.

## 5. Conclusions

Atherosclerosis is a dynamic disease that impacts various individuals differently. It can remain stable for years and at a certain point it starts progressing, or vice versa. It manifests with peaks of instability that we observe in one arterial system but not in another. As a result, while we already understand some aspects and mechanisms of atherosclerosis’ natural history, we still have a limited knowledge of the actual connections between what we consider the same condition in different districts.

## Figures and Tables

**Table 1:** Summary of optimal antithrombotic and plaque stabilization strategies for clinically relevant atherosclerosis in different arterial systems. Of note, these are chronic long-term default strategies—there are other recommendations for patients requiring anticoagulants for other indications or if they received recent percutaneous or surgical revascularization. According to the recent guidelines [21,22,23]. Green signifies a strong recommendation, yellow means that “it may be considered”, and red represents “no benefit”.

	CAD		Carotid Disease		PAD	
Antithrombotic therapy	Symptomatic (Secondary prevention)	Asymptomatic (Primary prevention)	Symptomatic	Asymptomatic	Symptomatic	Asymptomatic
	Aspirin long term	Aspirin for primary prevention in higher ASCVD risk	Aspirin or clopidogrel	No	Aspirin and low-dose rivaroxaban	No data
Statin	Yes	Yes	Yes	Yes	Yes	
First-line treatment of associated hypertension	Beta-blockers ACEIs and ARBs		ACEIs and ARBs		ACEIs and ARBs	

CAD = coronary artery disease; PAD = peripheral artery disease; ASCVD = Atherosclerotic Cardiovascular Disease. The colors represent the level of indication and evidence, according to the cited guidelines.

## Data Availability

Not applicable.

## Abbreviations

Coronary artery disease, CAD; low-density lipoprotein, LDL; thin-cap fibroatheroma, TCFA; peripheral artery disease, PAD; vascular smooth muscle cell, VSMC.
